# Effects of Occasional and Habitual Wearing of High-Heeled Shoes on Static Balance in Young Women

**DOI:** 10.3389/fspor.2022.760991

**Published:** 2022-03-30

**Authors:** Ayano Yamada-Yanagawa, Shun Sasagawa, Kimitaka Nakazawa, Naokata Ishii

**Affiliations:** ^1^Department of Life Sciences, Graduate School of Arts and Sciences, The University of Tokyo, Tokyo, Japan; ^2^Department of Human Sciences, Kanagawa University, Yokohama, Japan

**Keywords:** high-heeled shoes, postural control, women, center-of-pressure (CoP), quiet standing

## Abstract

The purpose of this study was to examine the effects of occasional and habitual wearing of high-heeled shoes on static balance in young women. Groups of habitual high-heel wearers and non-wearers (*n* = 7 in both groups) were asked to stand quietly on a force platform without shoes (WS condition) or with high heels (heel area 1 cm^2^, heel height 7 cm) (HH condition). During the trials, the center-of-pressure (CoP) position in the anterior-posterior direction was measured, and its root mean square (as a measure of postural sway magnitude, CoP_RMS_) and mean velocity (as a measure of regulatory activity, CoP_MV_) were calculated. To further examine the effect of high-heel wearing on the temporal aspects of slow and fast processes in static balance, the CoP sway was decomposed into low- (below 0.5 Hz) and high- (above 0.5 Hz) frequency components, and then spectral analysis was performed. Results showed that the CoP_RMS_ was not significantly different between the groups or between the shoe conditions, indicating that wearing high heels with a heel height of 7 cm did not increase the magnitude of postural sway, irrespective of high-heel experience. The CoP_MV_ was significantly larger in the HH condition than in the WS condition, whereas it was not significantly different between the groups. This result indicates that wearing high heels increased the amount of regulatory activity in both habitual wearers and non-wearers. The spectral analysis further showed that habitual high-heel wearers showed significantly decreased rate of regulatory activity than non-wearers, both while standing with and without high heels. These results suggest that use-dependent changes in static balance control are evident in both high-heeled and without shoes conditions.

## Introduction

In the mid-nineteenth century, high-heeled shoes became popular among women in all socioeconomic classes in many countries. During this period, women wore high heels to prevent their dresses dragging on the ground (Danesi, 2018). Although high heels are sometimes uncomfortable and unsuitable for locomotion, millions of women currently wear high heels in their daily lives. An important perceived benefit of wearing high heels is increased attractiveness of the wearer. Lewis et al. (2017) demonstrated that, when participants stood still in high heels, their lumbar curvature increased, and they were perceived as more attractive. Regarding walking, Morris et al. (2013) reported that walking in high heels enhanced the perceived femininity of gait by reducing stride length and increasing rotation and tilt of the hips.

However, various negative outcomes have been reported to be associated with habitual wearing of high heels, including orthopedic problems such as hallux valgus (Menz and Morris, 2005), knee osteoarthritis (Kerrigan et al., 2005), and lower back pain (Lee et al., 2001). In addition, regular use of high heels can induce structural and functional changes in the calf muscle-tendon unit (MTU), with studies reporting reduced fascicle length of gastrocnemius medialis (Csapo et al., 2010; Cronin et al., 2012), increased cross-sectional area and stiffness of the Achilles' tendon (Csapo et al., 2010), and reduced active range of motion of the ankle (Csapo et al., 2010) among women who regularly wear high heels. Recent studies have reported that functional interactions between muscle fibers and tendinous tissues are relevant not only for storing/releasing energy during dynamic movements (Fukunaga et al., 2002) but also for controlling static balance (Loram et al., 2005a). Therefore, changes in structural and mechanical properties of the calf MTU induced by long-term use of high heels are expected to result in substantial changes in the control of static balance. Some previous studies have investigated the effects of high-heel experience on static balance by comparing center-of-pressure (CoP) sway between habitual high-heel wearers and non-wearers while wearing high heels with different heel heights (Hapsari and Xiong, 2016; Wan et al., 2019). Wan et al. (2019) reported that the habitual high-heel wearers exhibited significantly smaller mean velocity of CoP (CoP_MV_) in the anterior-posterior (AP) direction for various heel heights (1, 5, 8, and 10 cm) compared with non-wearers. It should be noted that these previous studies compared static balance between habitual high-heel wearers and non-wearers while participants stood with heeled shoes. However, in the current study, we hypothesized that changes in static balance would become more prominent when standing barefoot or without shoes, because the reduced fascicle length of the calf muscle leads the resting positions of the ankle to be more plantarflexed (Csapo et al., 2010) and regular high-heel wearers often experience calf muscle pain when standing barefoot (Opila et al., 1988). Therefore, we examined this hypothesis by comparing CoP sway between habitual high-heel wearers and non-wearers while standing with and without high heels.

To quantify the CoP sway, two conventionally used statistical measures were calculated: the root mean square (CoP_RMS_) and CoP_MV_. CoP_RMS_ represents sway magnitude, or in other word, the effectiveness of postural control system, whereas CoP_MV_ represents the amount of regulatory activity (Hufschmidt et al., 1980; Prieto et al., 1996). Therefore, participants who exhibit slow and large drifts in the body position would have a large CoP_RMS_. In contrast, those who make more fast ballistic corrections of balance would have a large CoP_MV_ (Kirshenbaum et al., 2001). It has also been shown that low- (CoP_LF_) and high- (CoP_HF_) frequency components of the CoP sway reflect different processes in the control of static balance (Zatsiorsky and Duarte, 2000; Loram et al., 2005b). The CoP_LF_ below 0.5 Hz corresponds to the horizontal projection of the center of body mass (CoM). On the other hand, the CoP_HF_ around 1 Hz corresponds to ballistic regulation of ankle joint torque by which the postural control system regulates the CoM movement. Therefore, to further examine the effects of occasional and habitual wearing of high-heeled shoes on the temporal aspects of two different processes in static balance, we decomposed the CoP sway into the CoP_LF_ and CoP_HF_, and then performed spectral analysis.

## Materials and Methods

### Participants

Because previous studies have reported significant effects of high-heel experience on postural control during quiet standing (Hapsari and Xiong, 2016; Wan et al., 2019), we expected substantially large interaction in regulatory activity between habitual high-heel wearers and non-wearers. When we conducted *a priori* power analysis using G^*^power (Faul et al., 2007), with the two-way mixed-design analysis of variance (ANOVA), 0.05 significance threshold, 0.8 detection power, and effect size of partial eta squared ($\eta_{P}^{2}$) = 0.26, the required total sample size was calculated to be ten. We recruited 14 young women aged 21–39 from our colleagues and students. The participants were divided into two equally sized groups: a habitual high-heel wearer group and a non-wearer group (*n* = 7 in both groups). Participants in the wearer group (mean ± standard deviation (SD): age 31.4 ± 6.7 years, height 161.1 ± 7.1 cm, weight 54.3 ± 14.5 kg) wore high heels with heel height > 5 cm at least 5 days per week over the previous 2 years or longer. In contrast, participants in the non-wearer group (age 28.6 ± 7.7 years, height 159.9 ± 5.0 cm, weight 52.1 ± 4.6 kg) wore high heels with heel height > 5 cm < 2 days per week over the previous 2 years or longer. Independent-samples *t*-tests revealed no significant differences in age, height, or weight between the two groups. All participants were healthy and had no history of traumatic injury or surgical operation. All participants gave written informed consent to participate in this study. The experimental procedures used were approved by the Ethics Committee on Human Experimentation at the Graduate School of Arts and Sciences, The University of Tokyo, in accordance with the Declaration of Helsinki (#13-56).

### Protocol

**Figure 1:**
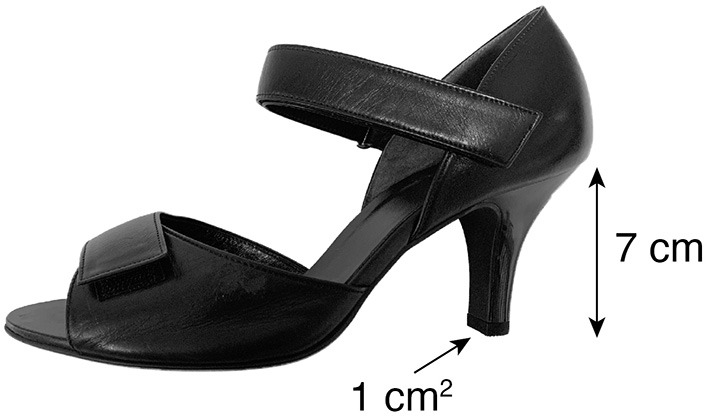
A side view of the custom-made high-heeled shoe (heel area 1 cm^2^, heel height 7 cm). The shoe had two adjustable straps with Velcro fasteners around the ankle and the dorsum of the foot.

To eliminate the effects of swelling of the feet and fatigue, all experiments were conducted in the morning or early afternoon. Custom-made high-heeled shoes (heel area 1 cm^2^, heel height 7 cm) in a range of sizes were prepared for this study (Figure 1). These high-heeled shoes had two adjustable straps with Velcro fasteners around the ankle and the dorsum of the foot. Prior to the experiment, a shoe-fitting session was performed to choose the best-fitting shoe size for each participant. In the shoe-fitting session and experiment, the tension of the shoe straps was adjusted by a professional shoe fitter (AY-Y). During a few minutes of shoe fitting and measurement preparation, the participants became accustomed to the standardized shoes. Participants were asked to wear stockings and stand quietly on a force platform (Type 9281B, Kistler, Switzerland) without shoes (WS condition) or while wearing high heels (HH condition) with their eyes open for just over 60 s. Participants held their arms comfortably by their sides, with their feet parallel to each other. Previous study demonstrated that narrow stance width (intermalleolar distance of <8 cm) increases postural sway in the mediolateral (ML) direction (Day et al., 1993). Therefore, we set the intermalleolar distance to 10 cm in order to minimize CoP sway in the ML direction. Five successive trials of the WS condition were followed by five successive trials of the HH condition. To avoid fatigue during the experiment, a rest of several tens of seconds was provided between trials in each shoe condition, and a rest of 5 min was provided between WS and HH conditions.

### Analysis

From the ground reaction forces measured by a force platform at a sampling frequency of 100 Hz, the CoP position was calculated. It has been reported that static balance in the AP direction is under the control of the ankle (plantar/dorsiflexor), whereas that in the ML direction is under the control of the hip (abductor/adductor) (Winter et al., 1996). Because we were interested in the effects of wearing of high heels on the ankle mechanism of static balance, CoP sway in the AP direction was measured and analyzed. The CoP time-series was digitally smoothed with a cut-off frequency of 3.0 Hz (Gage et al., 2004) using a built-in low-pass filter in commercial software (LabChart 7, AD Instruments, Australia). For the subsequent analysis, 60 s of data in the middle portion of the recorded data were used. From the CoP time-series, the CoP_RMS_ and CoP_MV_ were calculated, as follows:


(1) $$CoP_{RMS}=\sqrt{\frac{\sum_{i=1}^{N}{\left(x_{i}-\tilde{x}\right)}^{2}}{N}}$$

(2) $$CoP_{MV}=\frac{\sum_{i=1}^{N=1}\left|x_{i+1}-x_{i}\right|}{T}$$

where *x*_*i*_ denotes the CoP position at the *i*-th instant (*i* runs from 1 to *N* − 1 or *N*), $\tilde{x}$ denotes the mean CoP position, *N* denotes the number of data points used in the analysis (*N* = 6,000), and *T* denotes the duration of each trial (*T* = 60 s). The CoP_RMS_ and CoP_MV_ were normalized by participants' body height (BH) (Cattagni et al., 2014; Oba et al., 2015).

The CoP time-series was decomposed into the CoP_LF_ and CoP_HF_. The CoP_LF_ time-series was calculated by low-pass filtering the CoP time-series with a cut off-frequency of 0.5 Hz (Caron et al., 1997; Loram and Lakie, 2002). By subtracting the CoP_LF_ time-series from that of the CoP, we obtained the CoP_HF_ time-series. We quantified the temporal aspects of the CoP_LF_ and CoP_HF_ time-series using the mean power frequency (MPF). In the spectral analysis, the CoP_LF_ and CoP_HF_ time-series for a single trial was detrended and then divided into five segments (20 s each) with 50% overlap. A fast-Fourier transform algorithm was applied to each segment to yield the power spectral density (PSD) after being passed through a Hamming window (*pwelch* function in MATLAB R2021a, MathWorks, USA). The PSDs of individual segments were ensemble-averaged into the function for a single trial. Note that our use of a 20 s data window resulted in the frequency resolution of 0.05 Hz. The MPF was calculated as follows:


(3) $$\begin{array}{l} MPF=\frac{\int f\cdot P}{\int P} \end{array}$$

where *f* and *P* denote the frequency and PSD, respectively.

Results are presented as mean and 95% confidence interval (CI) in the text and as mean and individual values in the figures. In the statistical analysis, differences between the groups and test conditions were examined using a two-way mixed-design ANOVA with repeated measures on the shoe condition (IBM SPSS Statistics 21, IBM, USA). The significance level was set at *P* < 0.05, and Bonferroni correction was used when necessary. The effect size was reported as $\eta_{P}^{2}$. According to Bakeman (2005), we defined an $\eta_{P}^{2}$ of 0.02 as small, one of 0.13 as medium, and one of 0.26 as large.

## Results

**Figure 2:**
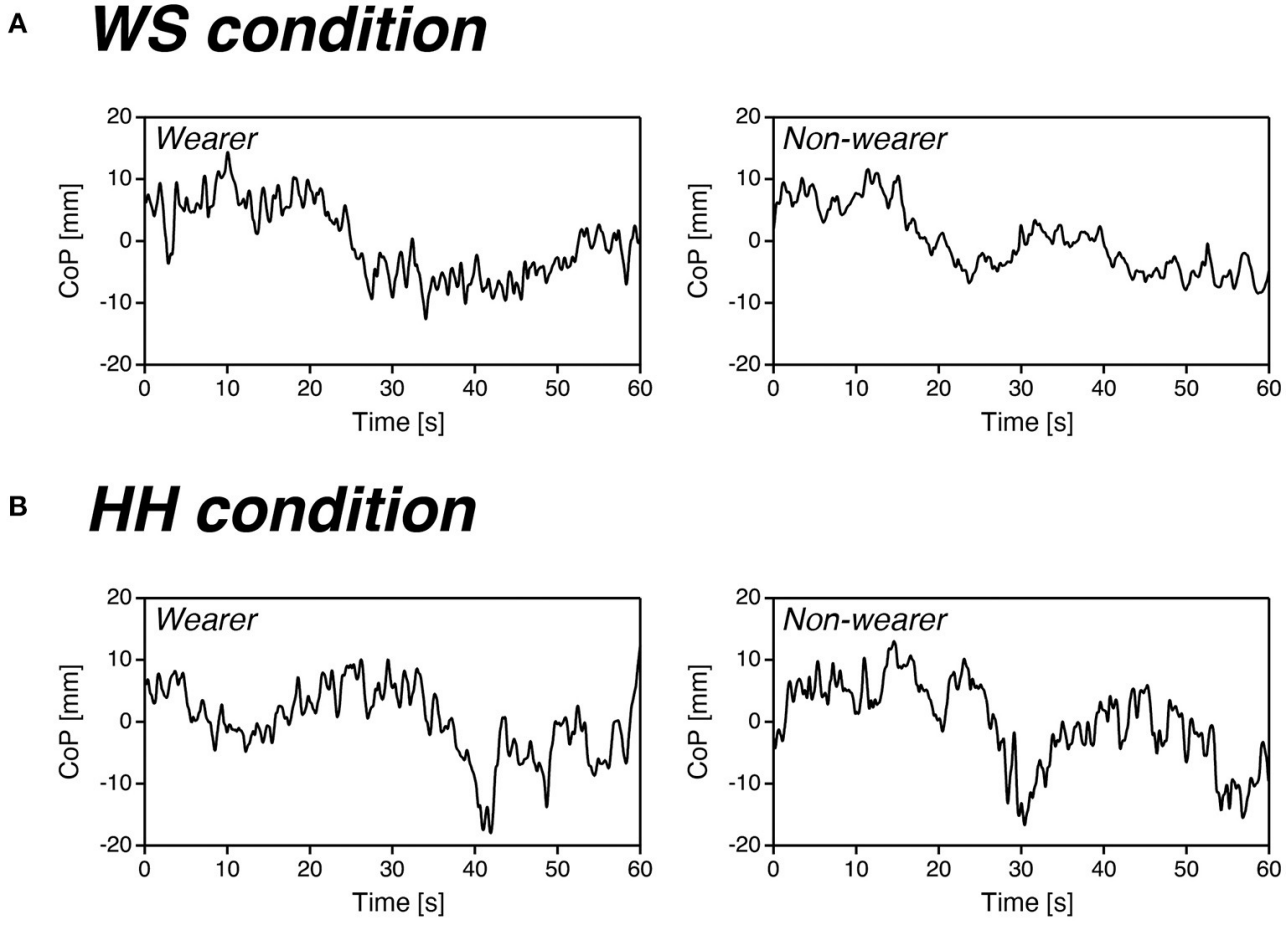
Representative recordings of the CoP sway for one high-heel wearer (*left*) and one non-wearer (*right*) in the WS **(A)** and HH **(B)** conditions. **(A)** Overall excursion of the CoP showed no difference between the two participants, whereas fast ripples were more prominent in the CoP recordings for the wearer. **(B)** Fast ripples were observed in the recordings for both the wearer and non-wearer, and no apparent differences were observed between the two participants.

Figure 2 shows representative recordings of the CoP sway for one habitual high-heel wearer (*left*) and one non-wearer (*right*) in the WS (*A*) and HH (*B*) conditions. It should be noted that these CoP recordings were presented with respect to their mean values. Several comparable features in these recordings should be noted. First, in the WS condition, the overall excursion of the CoP appeared to be no different between the wearer and non-wearer, whereas the fast ripples (~1 s per cycle) were more prominent in the CoP recordings for the wearer. In contrast to the WS condition, these fast ripples were observed in the recordings for both the wearer and non-wearer in the HH condition, and no apparent difference was observed between the two recordings.

**Figure 3:**
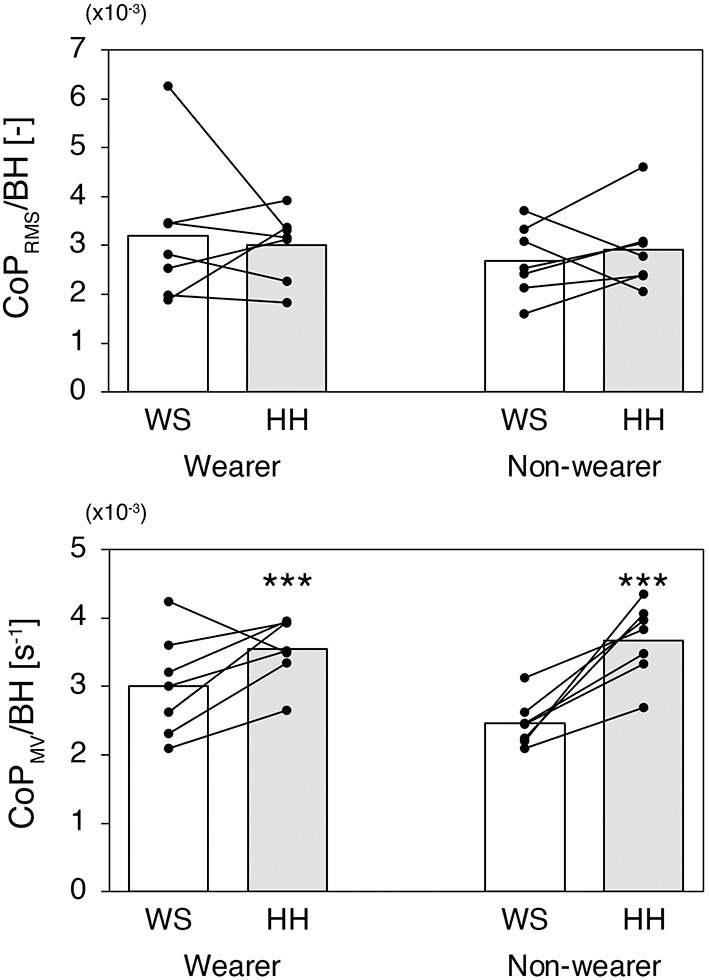
The CoP_RMS_ (*top*) and CoP_MV_ (*bottom*) for each group and each test condition. The white bar indicates the WS condition and the gray bar indicates the HH condition. The small dots represent data for individual participants. ^***^P < 0.001 between the test conditions.

Figure 3 shows results for the CoP_RMS_ (*top*) and CoP_MV_ (*bottom*) for each group and each test condition. For the CoP_RMS_, there was no significant difference between the wearer group (mean = 3.098×10^−3^, 95% CI [2.434×10^−3^, 3.762×10^−3^]) and non-wearer group (mean = 2.797×10^−3^, 95% CI [2.134×10^−3^, 3.461×10^−3^]) [*F*_(1,12)_ = 0.487, *P* = 0.499, $\eta_{P}^{2}$ = 0.039] or between the WS condition (mean = 2.942×10^−3^, 95% CI [2.259×10^−3^, 3.625×10^−3^]) and HH condition (mean = 2.953×10^−3^, 95% CI [2.501×10^−3^, 3.406×10^−3^]) [*F*_(1,12)_ = 0.001, *P* = 0.973, $\eta_{P}^{2}$ < 0.001]. The CoP_MV_ was significantly larger in the HH condition (mean = 3.611×10^−3^, 95% CI [3.313×10^−3^, 3.909×10^−3^]) than in the WS condition (mean = 2.735×10^−3^, 95% CI [2.393×10^−3^, 3.076 ×10^−3^]) [*F*_(1,12)_ = 27.800, *P* < 0.001, $\eta_{P}^{2}$ = 0.698], whereas there was no significant difference between the wearer group (mean = 3.280×10^−3^, 95% CI [2.906×10^−3^, 3.654×10^−3^]) and non-wearer group (mean = 3.065×10^−3^, 95% CI [2.691×10^−3^, 3.439×10^−3^]) [*F*_(1,12)_ = 0.790, *P* = 0.392, $\eta_{P}^{2}$ = 0.062]. There was no significant interaction between group and test condition [*F*_(1,12)_ = 4.127, *P* = 0.065, $\eta_{P}^{2}$ = 0.256].

**Figure 4:**
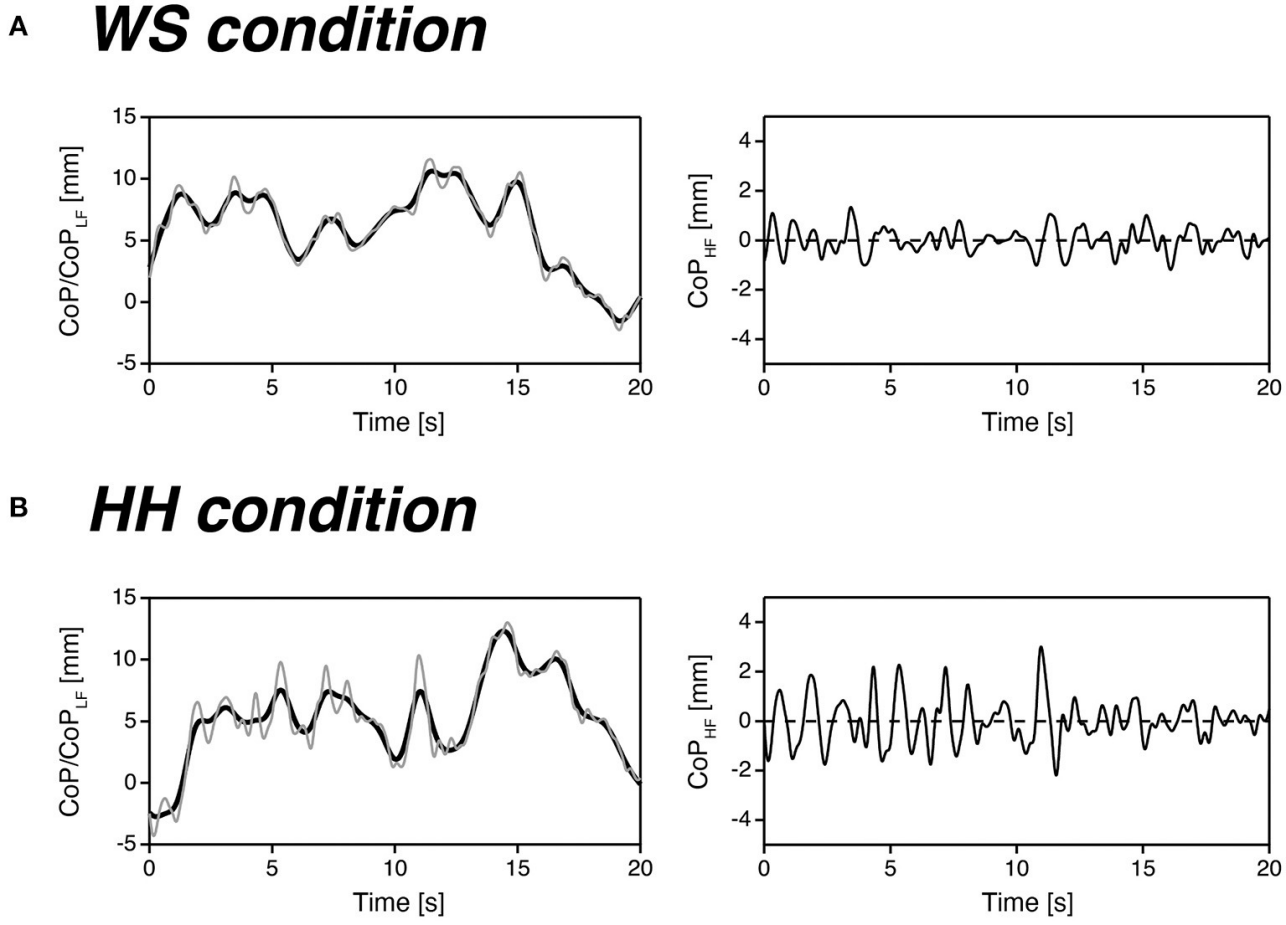
Representative examples of the CoP (*thin gray line*) and CoP_LF_ (*bold black line*) recordings (*left*) and corresponding CoP_HF_ recordings (*right*) for one non-wearer in the WS **(A)** and HH **(B)** conditions.

Figure 4 shows representative examples of the CoP (*thin gray line*) and CoP_LF_ (*bold black line*) recordings (*left*) and corresponding CoP_HF_ recordings (*right*) for one non-wearer (the same participant and trials as shown in Figure 2) in the WS (*A*) and HH (*B*) conditions. Note that only 20 s of data from the 60-s recordings are presented in this figure to emphasize the details of the waveforms. From these two CoP_HF_ recordings, it can be seen that this participant exhibited increased regulatory activity while standing with high heels.

**Figure 5:**
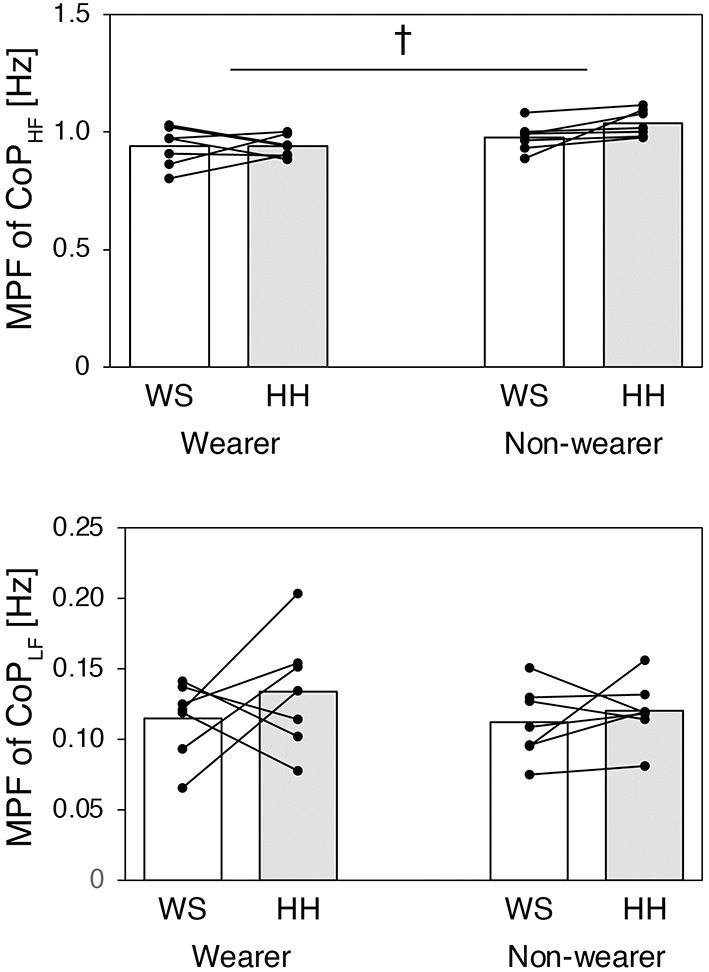
The MPF of CoP_HF_ (*top*) and CoP_LF_ (*bottom*) for each group and each test condition. The white bar indicates the WS condition and the gray bar indicates the HH condition. The small dots represent data for individual participants.^†^*P* < 0.05 between the groups.

Figure 5 shows results for the MPF of CoP_HF_ (*top*) and CoP_LF_ (*bottom*) for each group and each test condition. The MPF of CoP_HF_ was significantly higher in the non-wearer group (mean = 1.008, 95% CI [0.968, 1.049]) than in the wearer group (mean = 0.939, 95% CI [0.898, 0.979]) [*F*_(1,12)_ = 7.048, *P* = 0.021, $\eta_{P}^{2}$ = 0.370], while there was no significant difference between the WS condition (mean = 0.959, 95% CI [0.916, 1.001]) and HH condition (mean = 0.989, 95% CI [0.958, 1.019]) [*F*_(1,12)_ = 1.907, *P* = 0.192, $\eta_{P}^{2}$ = 0.137]. There was no significant interaction between group and test condition in the MPF of CoP_HF_ [*F*_(1,12)_ = 1.942, *P* = 0.189, $\eta_{P}^{2}$ = 0.139]. For the MPF of CoP_LF_, there was no significant difference between the wearer group (mean = 0.124, 95% CI [0.107, 0.141]) and non-wearer group (mean = 0.116, 95% CI [0.099, 0.133]) [*F*_(1,12)_ = 0.565, *P* = 0.467, $\eta_{P}^{2}$ = 0.045] or between the WS condition (mean = 0.113, 95% CI [0.098, 0.129]) and HH condition (mean = 0.127, 95% CI [0.108, 0.146]) [*F*_(1,12)_ = 1.418, *P* = 0.257, $\eta_{P}^{2}$ = 0.106].

## Discussion

### Differences in the CoP_RMS_ and CoP_MV_

To examine the effects of occasional and habitual wearing of high heels on static balance, we first compared CoP sway during quiet standing between habitual high-heel wearers and non-wearers. The results revealed no significant differences in CoP_RMS_ between groups, or between test conditions (Figure 3, *top*). Given that CoP_RMS_ represents the sway magnitude, this result indicates that wearing high heels with a heel height of 7 cm did not increase the magnitude of postural sway, irrespective of high-heel experience. In addition, the results revealed that CoP_MV_ was significantly greater in the HH condition compared with the WS condition (Figure 3, *bottom*), suggesting that participants exhibited more and/or strong regulatory changes in ankle joint torque while standing with high heels. It should be noted that the two-way ANOVA revealed a marginal significance for an interaction between the group and test condition in the CoP_MV_ (*P* = 0.065). Because of the small sample size of the present study (*n* = 7 each group), there may be a concern about making a Type II error by wrongly accepting a false null hypothesis. However, when we conducted *post-hoc* power analysis with $\eta_{P}^{2}$ = 0.256 and correlation among repeated measures = 0.265, the power was calculated to be 0.913. This result indicates that there was enough statistical power in the present study.

### Increased CoP_MV_ in Habitual High-Heel Wearers

Although it failed to reach significance as stated above, the habitual wearers tended to exhibit marginally greater CoP_MV_ in the WS condition. Specifically, CoP_MV_ in the HH condition was almost the same for the wearers and non-wearers, whereas that in the WS condition is about 20% larger for the habitual wearers (Figure 3, *bottom*). Use-dependent changes in the structural and functional properties of the calf MTU may be responsible for the increased CoP_MV_ found in habitual high-heel wearers. First, because the reduced fascicle length of the calf muscle induced by the long-term use of high heels leads the resting positions of the ankle to be more plantarflexed (Csapo et al., 2010), greater passive plantarflexion torque was expected to be exhibited in these participants in the anatomical ankle position (i.e., while standing without shoes). Changes in passive plantarflexion torque per unit displacement of the ankle joint angle were also expected to be greater in high-heel wearers because of the non-linearity of the tendon stiffness (Kubo et al., 2001). Second, greater tendon stiffness strengthens the mechanical coupling between the contraction forces of the muscle fibers and the reaction force acting on the ground (Lakie et al., 2003). Finally, it has been reported that habitual high-heel wearers exhibit greater maximal voluntary isometric plantarflexion torque (~10%) compared with non-wearers, at a wide range of ankle positions (−20° to +20°) (Csapo et al., 2010). All of these factors may contribute to amplification of the regulatory modulation of the ankle joint torque in habitual high-heel wearers, thereby increasing their CoP_MV_.

Increased CoP_MV_ during quiet standing has commonly been observed in studies of people aged 60 years and above (Prieto et al., 1996; Kouzaki and Masani, 2012). Moreover, increased amplitude of the fast component of CoP sway has been reported to be associated with increased risk of falls in older people (Collins et al., 1995; Piirtola and Era, 2006). Although the underlying physiological mechanisms may differ between older people and young habitual high-heel wearers, the increased CoP_MV_ may represent an unfavorable change in balancing strategy, such as in terms of energy efficiency. To minimize the potential negative effects of long-term use of high heels, habitual wearers are recommended to perform stretching routines after wearing high heels to maintain flexibility of the calf MTU.

### Differences in the MPF of CoP_HF_ and CoP_LF_

Frequency domain analysis on the CoP_HF_ revealed that habitual high-heel wearers exhibited significantly lower MPF than non-wearers, regardless of the test conditions (Figure 5, *top*). This result indicates that habitual wearers showed decreased rate of regulatory activity than non-wearers, both while standing with and without high heels. Interestingly, occasional wearing of high heels by novice wearers resulted in an opposite effect on the MPF of CoP_HF_. That is, although not statistically significant, the MPF of CoP_HF_ in novice wearers was slightly increased in the HH condition (mean ± SD: WS condition 0.98 ± 0.06 Hz, HH condition 1.04 ± 0.06 Hz). It is also interesting to note that, in habitual wearers, the MPF of CoP_HF_ remained almost unchanged in the WS and HH conditions (WS condition 0.94 ± 0.08 Hz, HH condition 0.94 ± 0.05 Hz), suggesting that long-term, habitual wearing of high-heeled shoes may change static balance control irrespective of shoe conditions. In contrast to the results for the MPF of CoP_HF_, there were no significant differences in the MPF of CoP_LF_ between groups or shoe conditions (Figure 5, *bottom*), indicating that occasional or habitual wearing of high heels has no effect on the temporal aspects of slow bodily sway.

### Biomechanical and Neurophysiological Effects of Wearing High Heels

Standing with high heels is similar to a condition in which participants stand over a declined surface (although some part of the forefoot is placed on a horizontal surface). Mezzarane and Kohn (2007) investigated the postural control on the inclined surfaces (14° toes-up, horizontal, and 14° toes-down) and found that declined surface significantly increased MPF of CoP and electromyography activity of soleus. Sasagawa et al. (2009b) also found increased tonic activities of soleus and gastrocnemius during toes-down standing and speculated that these increased tonic activities play a role in enhancing the ankle stiffness. With regard to the high-heel wearing, especially in non-wearers, it is likely that the posterior calf muscles increase tonic activity to eliminate the slack in the Achilles' tendon and to enhance ankle stiffness. Furthermore, high-heel wearing can affect the neurophysiological properties of the anterior calf muscles. For example, because the ankle joint is plantarflexed during standing with high-heels, increased Ia discharges is expected from the stretched anterior calf muscles. Loram and colleagues (Di Giulio et al., 2009; Loram et al., 2009) have suggested that passive and unmodulated anterior calf muscle (e.g., tibialis anterior), uncomplicated by fluctuations in muscle activity, enables a better proprioception of small, joint rotation during quiet standing. Although it is uncertain whether the expected increase in Ia discharges from the anterior calf muscles interferes with proprioception as mere background noise or enhances it via a mechanism known as stochastic resonance (Cordo et al., 1996), high-heel wearing may have a certain influence on the proprioception of postural sway.

### Comparison With Previous Studies

Hapsari and Xiong (2016) examined the effects of heel height and high-heel experience on static balance, reporting that wearing high heels with a heel height up to 7 cm did not impair postural stability, irrespective of high-heel experience. Similarly, Wan et al. (2019) reported no significant differences in CoP_RMS_ among four different heel conditions (1, 5, 8, and 10 cm) when habitual wearers and non-wearers were analyzed together. In addition, the authors reported no significant differences in CoP_RMS_ between high-heel wearers and non-wearers in 1 and 5 cm heel conditions (Wan et al., 2019). These CoP_RMS_ results are consistent with the current findings. However, there are several discrepancies in CoP_MV_ results between studies. For example, Wan et al. (2019) demonstrated that CoP_MV_ decreased as heel height increased from 1 to 8 cm. In addition, high-heel wearers were reported to exhibit significantly smaller CoP_MV_ in all heel conditions compared with non-wearers (Wan et al., 2019). The difference in the mean age of participants could have potentially caused the discrepancies found between studies. In particular, because the high-heel wearers in Wan et al.'s study were relatively young (mean ± SD: 24.6 ± 2.1 years), it is possible that these participants were not fully accustomed to wearing high heels (Wan et al., 2019). Therefore, the results reported by Wan et al. (2019) may reflect the transition process of becoming accustomed to wearing high heels. The high-heel wearers recruited in the present study were substantially older (31.4 ± 6.7 years) than those in Wan et al.'s study (Wan et al., 2019). Thus, it is likely that most of the high-heel wearers in the present study were fully accustomed to wearing high heels. Differences in the methods of measurement and analysis may have also contributed to the discrepancies between the studies. For example, Wan et al. (2019) measured the CoP position using an insole measuring system, whereas we measured it using a standard force platform system. Furthermore, the previous study did not perform any filtering before calculating the CoP_MV_. This may have resulted in the large values of CoP_MV_ reported in that study (~20 mm/s). The CoP_MV_ of healthy young adults during quiet standing was reported to be around 5–6 mm/s (Prieto et al., 1996; Ushiyama and Masani, 2011), which is comparable to the values observed in the present study (note that unnormalized data are not reported in the present study).

### Limitations and Future Research

It should be noted that the CoP position in the AP direction is proportional to the ankle plantarflexion torque (Winter et al., 1998). Therefore, analyses of CoP sway only provide information regarding control around the ankle joint. Although the ankle joint plays a crucial role in controlling static balance (Winter et al., 1998; Masani et al., 2003), several recent studies have indicated significant contributions of the proximal joints (e.g., the hip and knee) even in an unperturbed stance (Sasagawa et al., 2009a, 2014; Yamamoto et al., 2015). In addition, Hapsari and Xiong (2016) reported that participants employed more hip strategy (less ankle strategy) to control static balance as the heel height of the shoes increased. Although the present study did not measure the CoP sway in the ML direction, this increased hip contribution may affect the postural sway in the ML direction. Therefore, to fully understand the changes in static balance control induced by occasional or habitual wearing of high heels, future studies should include three-dimensional, whole-body motion analysis and pay attention to multi-joint coordination.

## Conclusions

In the present study, we examined the effects of occasional and habitual wearing of high heels on static balance by comparing the CoP sway between high-heel wearers and non-wearers while standing with and without high heels. We first found that wearing high heels with a heel height of 7 cm did not increase the magnitude of postural sway, irrespective of high-heel experience. We also found that wearing high heels with heel height of 7 cm increased the amount of regulatory activity in both high-heel wearers and non-wearers. We further found that habitual high-heel wearers showed decreased rate of regulation of the ankle joint torque than non-wearers, both while standing with and without high heels. These results suggest that use-dependent changes in static balance control are evident in both high-heeled and without shoes conditions.

## Data Availability Statement

The original contributions presented in the study are included in the article/supplementary material, further inquiries can be directed to the corresponding author.

## Ethics Statement

The studies involving human participants were reviewed and approved by Ethics Committee on Human Experimentation at the Graduate School of Arts and Sciences, the University of Tokyo. The patients/participants provided their written informed consent to participate in this study.

## Author Contributions

AY-Y and NI: conceptualization. AY-Y: investigation. AY-Y and SS: formal analysis and writing—original draft preparation. AY-Y, SS, KN, and NI: writing—review and editing. All authors contributed to the article and approved the submitted version.

## Conflict of Interest

The authors declare that the research was conducted in the absence of any commercial or financial relationships that could be construed as a potential conflict of interest.

## Publisher's Note

All claims expressed in this article are solely those of the authors and do not necessarily represent those of their affiliated organizations, or those of the publisher, the editors and the reviewers. Any product that may be evaluated in this article, or claim that may be made by its manufacturer, is not guaranteed or endorsed by the publisher.
